# TMT-based proteomic analysis of liquorice root in response to drought stress

**DOI:** 10.1186/s12864-022-08733-z

**Published:** 2022-07-19

**Authors:** Dong Zhang, Zhongren Yang, Xiaoqing Song, Fenglan Zhang, Yan Liu

**Affiliations:** 1 College of Horticultural and Plant Protection, Inner Mongolia Agricultural University, Hohhot, 010011 China; 2 Inner Mongolia Key Laboratory of Wild Peculiar Vegetable Germplasm Resource and Germplasm Enhancement, Inner Mongolia Agricultural University, Hohhot, 010011 China

**Keywords:** Differential expression protein, Drought stress, Liquorice, Proteomics, TMT labeling

## Abstract

**Background:**

Drought stress is a serious threat to land use efficiency and crop yields worldwide. Understanding the mechanisms that plants use to withstand drought stress will help breeders to develop drought-tolerant medicinal crops. Liquorice (*Glycyrrhiza uralensis* Fisch.) is an important medicinal crop in the legume family and is currently grown mostly in northwest China, it is highly tolerant to drought. Given this, it is considered an ideal crop to study plant stress tolerance and can be used to identify drought-resistant proteins. Therefore, to understand the effects of drought stress on protein levels of liquorice, we undertook a comparative proteomic analysis of liquorice seedlings grown for 10 days in soil with different relative water content (SRWC of 80%, 65%, 50% and 35%, respectively). We used an integrated approach of Tandem Mass Tag labeling in conjunction with LC–MS/MS.

**Results:**

A total of 7409 proteins were identified in this study, of which 7305 total proteins could be quantified. There were 837 differentially expressed proteins (DEPs) identified after different drought stresses. Compared with CK, 123 DEPs (80 up-regulated and 43 down-regulated) were found in LS; 353 DEPs (254 up-regulated and 99 down-regulated) in MS; and 564 DEPs (312 up-regulated and 252 down-regulated) in SS.The number of differentially expressed proteins increased with increasing water stress, and the number of up-regulated proteins was higher than that of down-regulated proteins in the different drought stress treatments compared with the CK. Used systematic bioinformatics analysis of these data to identify informative proteins we showed that osmolytes such as cottonseed sugars and proline accumulated under light drought stress and improved resistance. Under moderate and severe drought stress, oxidation of unsaturated fatty acids and accumulation of glucose and galactose increased in response to drought stress. Under moderate and severe drought stress synthesis of the terpene precursors, pentacene 2,3-epoxide and β-coumarin, was inhibited and accumulation of triterpenoids (glycyrrhetinic acid) was also affected.

**Conclusions:**

These data provide a baseline reference for further study of the downstream liquorice proteome in response to drought stress. Our data show that liquorice roots exhibit specific response mechanisms to different drought stresses.

**Supplementary Information:**

The online version contains supplementary material available at 10.1186/s12864-022-08733-z.

## Background

As a result of climate change, drought stress has become one of the most common abiotic stresses affecting plant growth and development [1]. Drought stress affects the physiological metabolism, yield and quality of crops [2]. The initial response of plants to drought stress is usually a change in gene expression [3], and these stress-induced changes in gene expression can alter the protein expression profile. Protein synthesis as a biochemical signal is related to abiotic (e.g. water, salinity and freezing) and biotic stresses (e.g. diseases, insects and damage), and has both a direct impact on plant growth and development, and also helps explain changes in metabolites that occur between transcription and production [4]. Drought stress-inducible proteins are broadly divided into two categories: functional proteins, which play a direct protective role in cells, including ion channel proteins, late embryogenesis abundance, Stress associated protein, oxygen-evolving enhancer protein, heat shock proteins and metabolic enzymes [5–7], and regulatory proteins, which are mainly involved in abiotic stress signal transduction or regulation of related gene expression, including protein kinases, calmodulin and some signalling factors, etc. [8]. ABA is a major plant hormone involved in the drought stress response of plants and is involved in stomatal closure and expression of stress-responsive genes [9]. for ABA to fulfil its intercellular messenger function, it must be rapidly transported to the xylem after synthesis in the root system and be able to rapidly reach the aboveground via transpiration flow along the xylem [10].TMT-based proteomics technology (Tandem Mass Tags, TMT) is an in vitro isotopically labeled relative and absolute quantification technique developed by Thermo scientific [11], which allows extensive screening of protein expression profiles and provides comprehensive information about individual proteins [12]. In recent years, proteomics techniques have been used to resolve the response mechanisms of many crops to drought stress e.g. wheat [13], cereals [14] and cotton [15] amongst others.

Liquorice (*Glycyrrhiza uralensis* Fisch.) is a perennial herb in the legume family Glycyrrhiza [16]. Liquorice is an important Chinese medicine and food but also, as a result of high levels of active compounds in its root system (e.g. glycyrrhizic acid, flavonoids) and its well-developed root network [17], it also has pharmacological activity [18], can be used as a sweetener [19], and has a role in ecological restoration [20] and wind and sand control [21].

Glycyrrhetinic acid content is an important parameter in determining the quality of liquorice, and its biosynthesis is regulated by various enzymes e.g. 3-hydroxy-3-methylglutary CoA reductase [22], squalene synthase [23] and β-coumarinol synthase [24]. In the terpene biosynthesis process the mevalonate (MVA) and deoxyxylulose-5-phosphate (DXP) or methylerythritol-4-phosphate (MEP) are the predominant pathways for synthesis of the five-carbon compounds that are precursor of terpenoids. The corresponding compounds are synthesized by redox, acylation and glycosylation reactions [25, 26].

Cultivated liquorice is predominantly distributed in northwest China, where poor soil conditions and low rainfall severely limit the flourishing liquorice industry. It has been shown that moderate drought stress (RSWC 45%-50%) is beneficial for synthesis and accumulation of glycyrrhetinic acid [27]. However, the molecular mechanisms underlying the root response of liquorice to different levels of drought are not clear, hindering the quality of cultivated liquorice. To this end, this experiment was done to determine differential protein expression profiles amongst liquorice roots exposed to different water stresses. We used quantitative proteomics with TMT tagging combined with liquid chromatography-tandem mass spectrometry (LC–MS/MS) to compare, analyze and screen the relevant differential proteins, elucidate protein expression and function, and inform future research to improve the quality of cultivated liquorice.

## Results

### Effect of drought stress on the growth index of liquorice

**Fig. 1:**
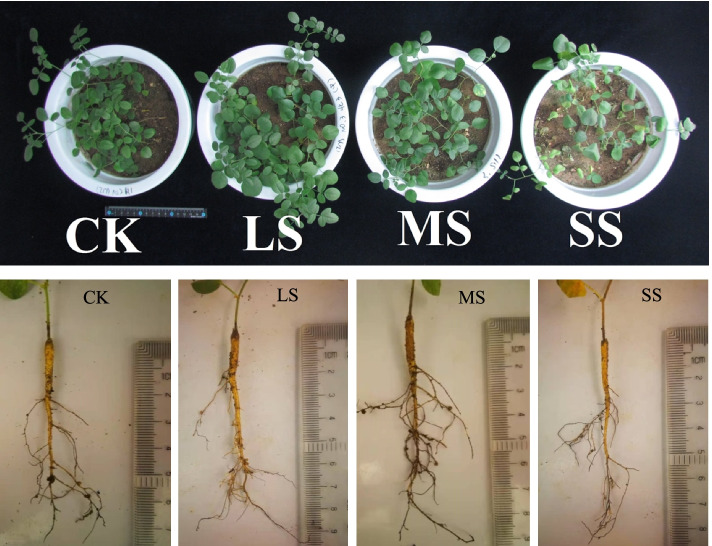
Liquorice morphology. Note: CK, LS, MS and SS represented normal water supply (CK), light drought stress (LS), moderate drought stress (MS) and severe drought stress (SS), respectively

Our experimental results showed that the shoot fresh weight, root fresh weight, plant height and root length of liquorice tended to increase and then decrease as the degree of drought stress increased, while the root-shoot ratio tended to increase gradually. After light stress (LS) and moderate stress (MS), shoot fresh weight, root fresh weight and root-shoot ratio were not significantly different from the control (CK), while plant height and root length were significantly different from CK; after severe stress (SS), shoot fresh weight, root fresh weight and root length were significantly lower than CK, while root-shoot ratio was significantly higher than CK (Fig. 1, Table 1).

**Table 1:** Changes in liquorice growth indicators under drought stress

	shoot fresh weight (g)	root fresh weight (g)	Plant height(cm)	Root length (cm)	root-shoot ratio
CK	0.58 ± 0.04 a	0.49 ± 0.06 ab	10.53 ± 0.50 b	7.67 ± 0.11 c	0.850 ± 0.14 b
LS	0.61 ± 0.06 a	0.52 ± 0.05 a	13.47 ± 0.50 a	9.03 ± 0.15 a	0.853 ± 0.07 b
MS	0.58 ± 0.02 a	0.54 ± 0.04 a	14.00 ± 1.00 a	8.63 ± 0.15 b	0.928 ± 0.10 ab
SS	0.40 ± 0.02 b	0.43 ± 0.04 c	9.33 ± 1.53 b	7.13 ± 0.15 d	1.07 ± 0.08 a

a, b, ab, c, d etc. in Table 1 represented that means in the same column followed by the same letter (a, b, c, d) do not differ significantly from each other according to Duncan’s test at 5% significance (*p* < 0.05), and the means in the same column followed by different letter differ significantly.. Each value indicates the treatment mean ± SE (*n* = 3)

### Effect of drought stress on photosynthetic enzyme activity of liquorice

**Fig. 2:**
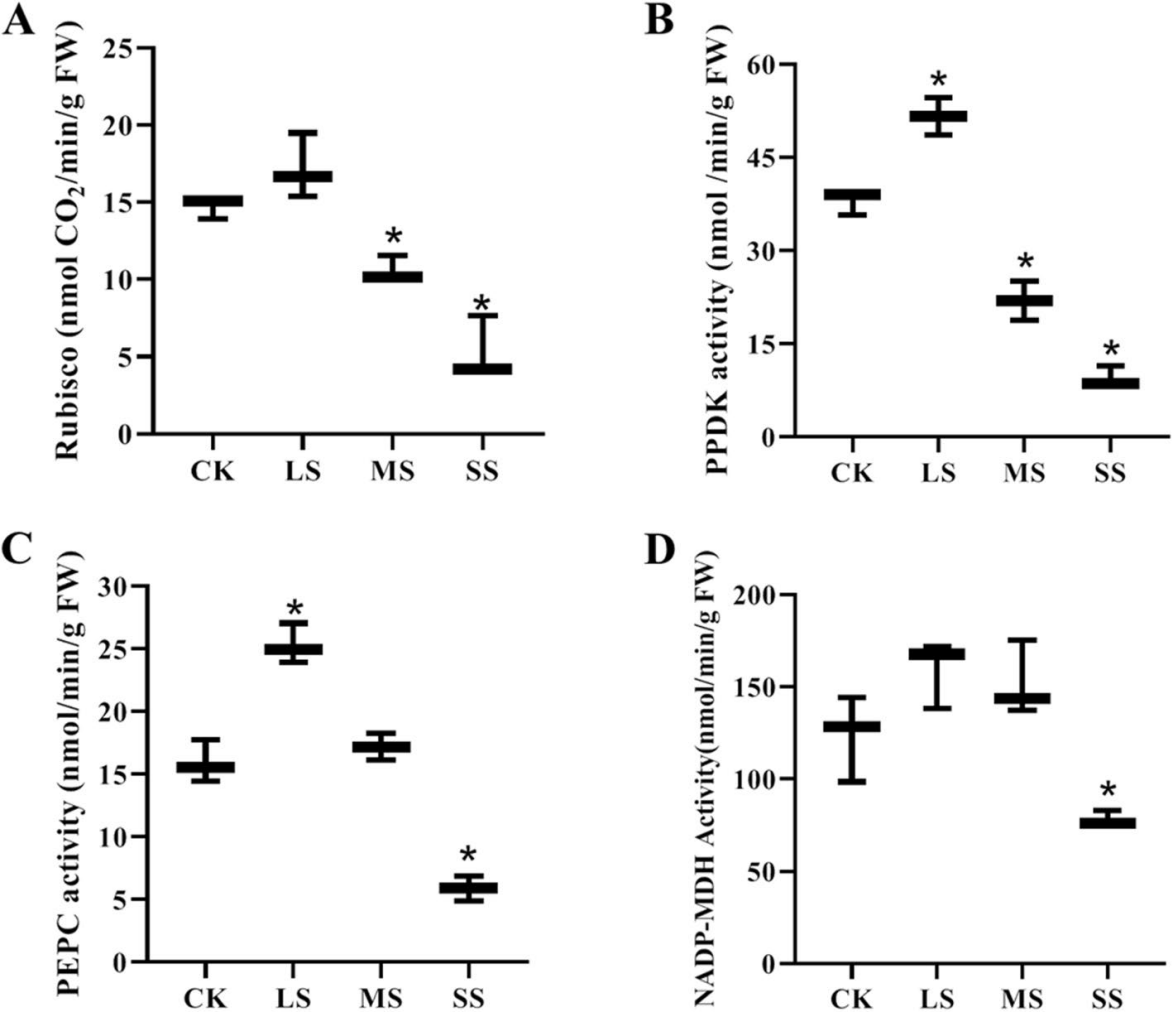
Changes of photosynthetic enzyme activity in liquorice leaves under drought stress. Note:CK, LS, MS and SS represented normal water supply (CK), light drought stress (LS), moderate drought stress (MS) and severe drought stress (SS), respectively; **A** Rubisco: ribulose 1,5-diphosphate carboxylase/oxygenase; **B** PPDK: pyruvate orthophosphate dikinase; **C **PEPC: phosphoenolpyruvate carboxylase; **D** NADP-MDH: NADP-Malate dehydrogenase

The activities of 1,5-diphosphate ribulose carboxylase(Rubisco), pyruvate phosphate double kinase(PPDK), phosphoenolpyruvate carboxylase(PEPC) and NADP-malate dehydrogenase(NADP-MDH) showed a trend of increasing and then decreasing with the increase of drought stress (Fig. 2). The activity of ADP-malate dehydrogenase increased by 16.52%, 36.38%, 58.9% and 28.67% respectively after light drought stress, while after severe drought stress it was significantly lower than that of the control, decreasing by 63.72%, 74.71%, 62.94% and 36.62% respectively.

### A general overview of protein identification in liquorice roots under drought stress

**Fig. 3:**
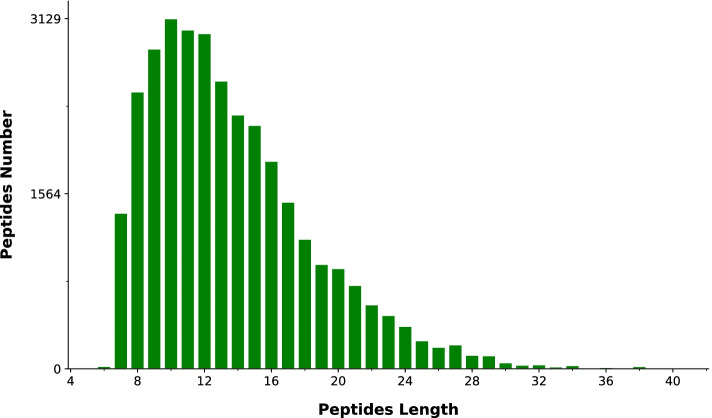
Distribution of peptide length range. Note: The x-axis indicates the peptides length, and the y-axis indicates peptides number

**Fig. 4:**
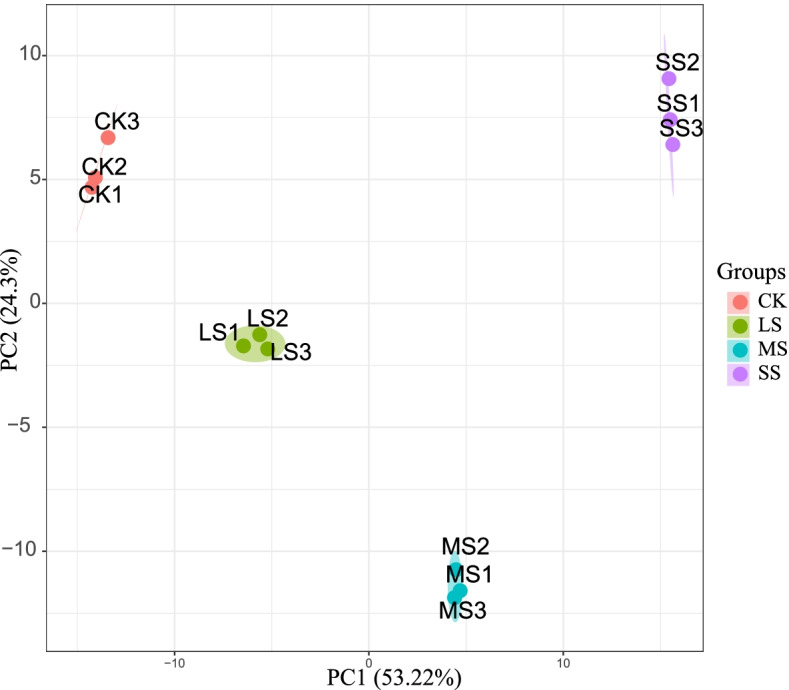
PCA analysis. Note: The horizontal coordinate PC1 and the vertical coordinate PC2 in the figure indicate the scores of the first and second ranked principal components, respectively. The scatter colour indicates the experimental grouping of the sample

A total of 430,807 secondary spectra, 32,537 matched spectra, 21,362 identified peptides and 7409 identified proteins were obtained by MS analysis; the total number of quantifiable proteins for all samples was 7305. The peptide length distribution analysis showed that most of the peptides were distributed in the range of 7–20 amino acids (Fig. 3), this was in accordance with the general pattern based on trypsin enzymatic digestion and HCD fragmentation mode and met quality control requirements. The results of principal component analysis showed that these 12 samples were clearly distinguished into four major categories marked with different colours in Fig. 4, indicating that protein expression had clear biological reproducibility.

### Quantification and annotation of differentially-expressed proteins (DEPs) in liquorice roots under drought stress

**Fig. 5:**
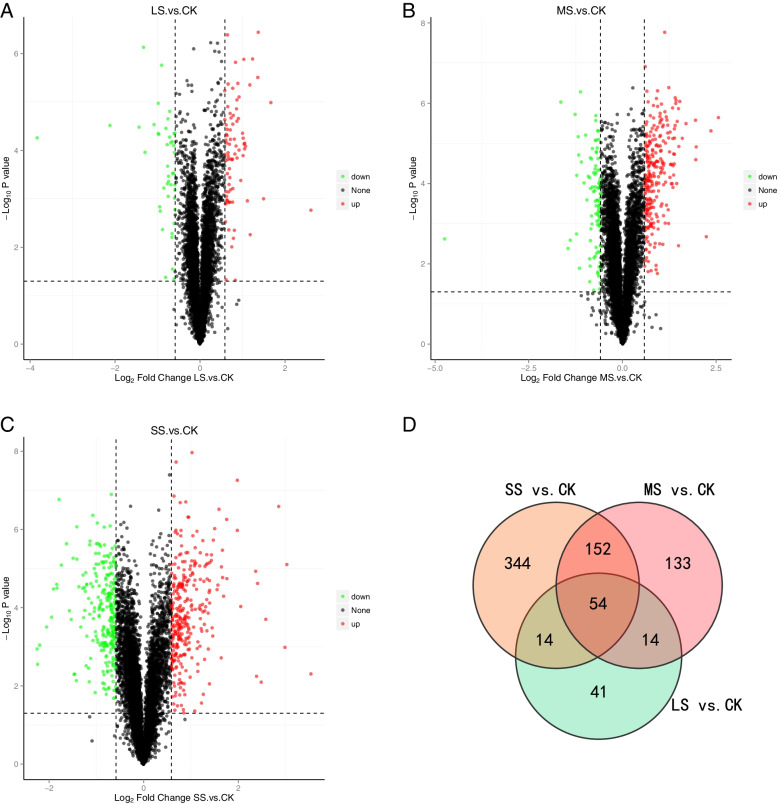
Volcano plot and Venn diagram of differential protein expression in liquorice root after different levels of water stress. Note: **a**, **b**, **c**: The horizontal coordinates indicate the difference multiplicity of differential proteins (log2 value), the vertical axis indicates the *P* value (-log10 value), black represents proteins that are not differentially expressed compared with the control; red represents up-regulated proteins; and green represents down-regulated proteins. **d**: Each circle in the graph represents a comparison group, and the numbers in the overlapping part of the circles represent the number of differentially-expressed proteins shared between the comparison groups; the numbers in the non-overlapping part represent the number of differentially-expressed proteins that are specific to that group

To understand in detail the differences between different drought treatments and controls, we compared LS, MS, and SS with CK (i.e. ‘LS vs. CK’, ‘MS vs. CK’, and ‘SS vs. CK’). A total of 837 differentially-expressed proteins were identified(Table S1). Of these, 35 DEPs were consistently up-regulated in expression and 19 DEPs were consistently down-regulated in expression as drought stress moved from LS to SS (Fig. 5.d).

The 35 up-regulated DEPs were involved in metabolic processes, redox processes, translational elongation, protein metabolic processes, carbohydrate metabolic processes, phosphatidylinositol phosphorylation, biosynthetic processes, cation transport and transmembrane transport.

The number of differentially expressed proteins increased with increasing water stress, and the number of up-regulated proteins was higher than the number of down-regulated proteins in the different drought stress treatments compared with the control. Compared with CK, 123 DEPs (80 up-regulated and 43 down-regulated) were found in LS (Fig. 5.a), 353 DEPs (254 up-regulated and 99 down-regulated) in MS (Fig. 5.b), and 564 DEPs (312 up-regulated and 252 down-regulated) in SS (Fig. 5.c).

### GO enrichment analysis of DEPs in liquorice roots under drought stress

**Fig. 6:**
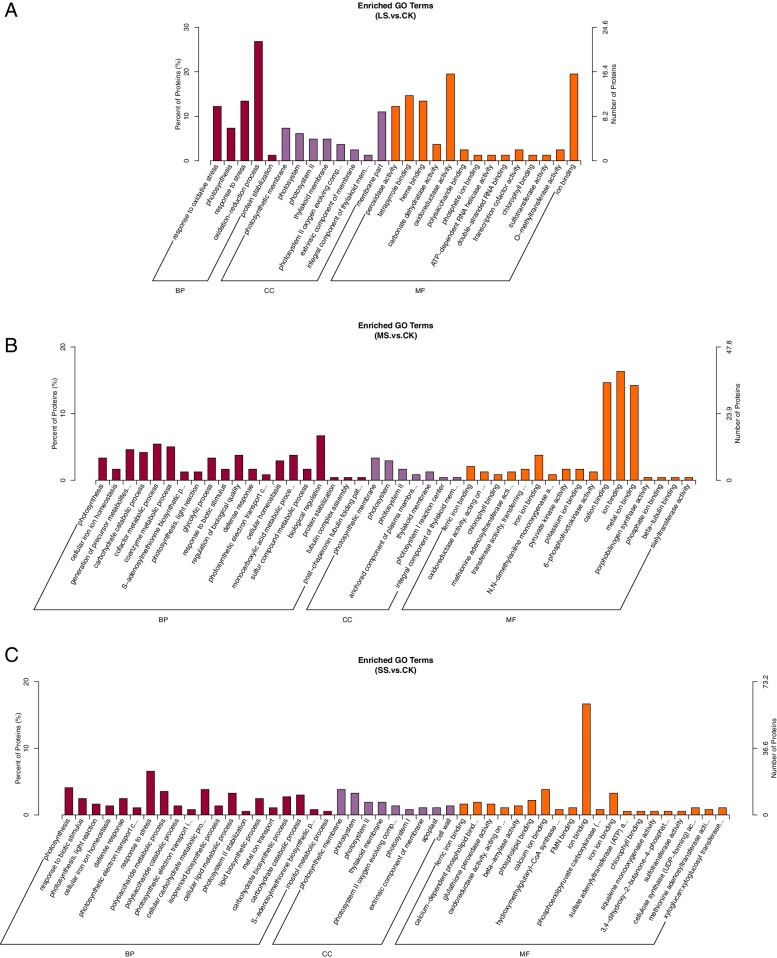
Bar chart of differential protein GO enrichment in liquorice roots from different comparison groups. Note: The figure shows the enrichment results in three categories, up to 20 of each (*P* value ≤ 0.05), and the percentages of the vertical coordinates represent x/n in the table

To resolve the response mechanisms of liquorice to different degrees of drought stress, we used GO analysis of the identified proteins (Fig. 6, Table S1). We found that the number of proteins in root tissues varied for each GO entry from LS, MS to SS.

Most of the processes showed an increase in the number of proteins, for example, the ‘membrane’ and ‘protein complex’ in the cellular component (CC); the ‘beta-amylase activity’ and ‘ion binding’ in the molecular function (MF); the ‘defense response’ in the biological process (BP).

### KEGG pathway enrichment of DEPs in liquorice roots under drought stress

**Fig. 7:**
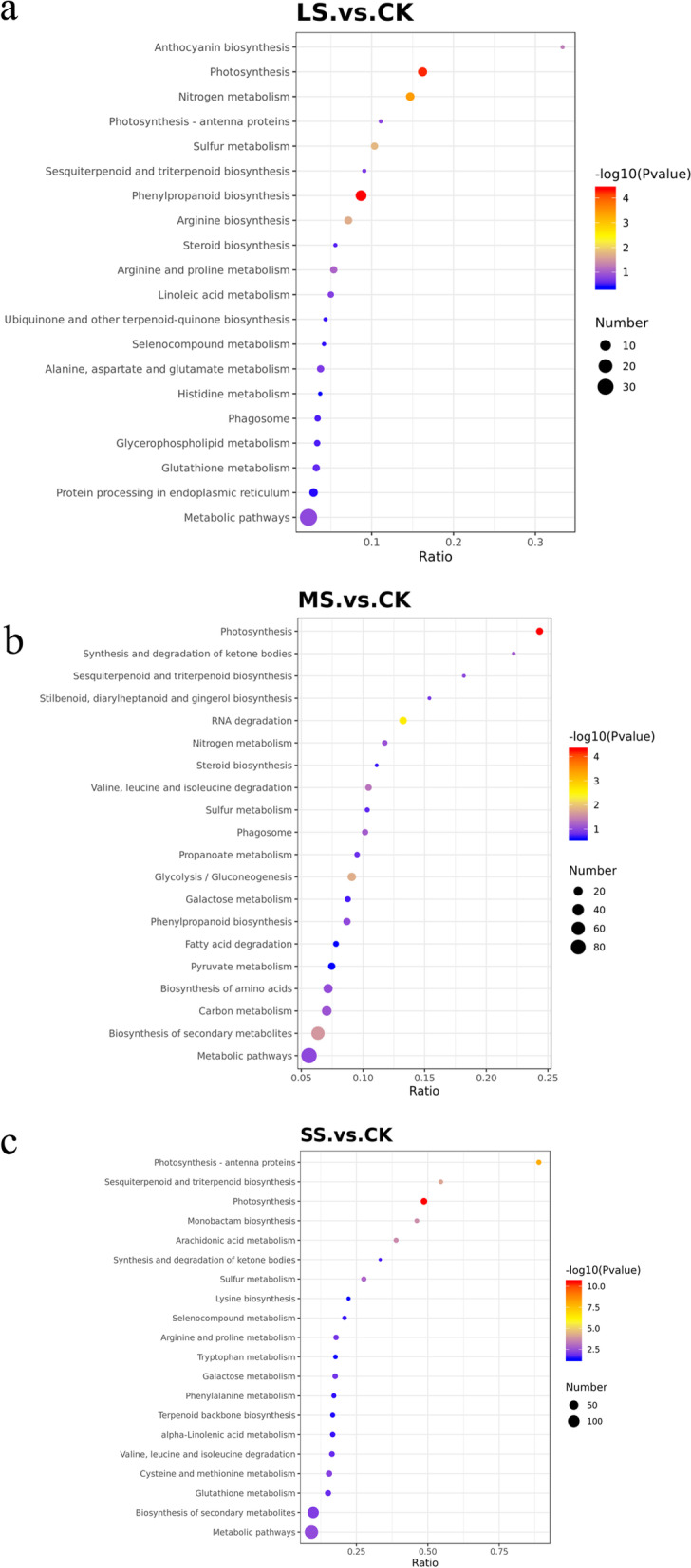
Bubbles representing different comparison groups of liquorice roots following differential protein KEGG enrichment. Note: The horizontal coordinate in the graph is the ratio of the number of differential proteins in the corresponding pathway to the number of total proteins identified in that pathway; the larger the value, the higher the differential protein enrichment in that pathway. The colour of the dot represents the *P* value of the hypergeometric test; the colours range from blue to red, the redder the colour, the smaller the value and the more reliable and statistically significant the test is. The size of the dots represents the number of differential proteins in the corresponding pathway, the larger the dot, the more differential proteins are present in the pathway

To further clarify the metabolic and signal transduction pathways involved in growth and development-related DEPs of liquorice roots under drought stress and in the control (CK) treatment, and to gain insight into the biological functions of DEPs, the DEPs obtained were subjected to KEGG metabolic pathway enrichment analysis(Fig. 7, Table S2). Compared with the control, 35 metabolic pathways were involved in LS, with most proteins involved in metabolic pathways (36), biosynthesis of secondary metabolites (21), biosynthesis of phenylpropanoids (10) and biosynthesis of amino acids (7); 53 pathways were involved in MS, with most proteins involved in metabolic pathways (89), biosynthesis of secondary metabolites (60), carbon metabolism (24), amino acid biosynthesis (21) and glycolysis/glyoxalate production (16); 55 pathways were involved in SS, with most proteins involved in metabolic pathways (146), biosynthesis of secondary metabolites (93), amino acid biosynthesis (28), glycolysis/glyoxalate production (20), photosynthesis (18), metabolism of cysteine and methionine (16), and protein processing in the endoplasmic reticulum (16). From above it could be seen that the number of proteins involved in the same metabolic pathway increased with increasing drought stress.

Also, we found that the photosynthesis pathway was significantly enriched in all three comparison groups. After LS, the proteins (PsbC, PsbH, PsbP, PsbQ, PsbR) in photosystem II were significantly down-regulated, and the enzymes of Calvin cycle (G7KMR3, large chain of ribulose bisphosphate carboxylase and small chain of ribulose bisphosphate carboxylase) were also down-regulated.After MS, photosystem I protein (PsaD), photosystem II protein (PsbH, PsbP, PsbO), cytochrome F (PetA, PetE) were significantly down-regulated. The photosynthetic carbon fixation pathway of glyceraldehyde phosphodehydrogenase (A0A1S2YBD0, A0A1S2XQX3) and ribulose diphosphate carboxylase small chain (G7KMR3) were significantly down-regulated. Malate dehydrogenase (A0A1S2XVZ6, A0A072TRS0) and fructose diphosphate aldolase (I1NFR3) were significantly up-regulated.After SS, Photosystem I proteins (PsaB, PsaD, PsaG), photosystem II proteins (PsbA, PsbB, PsbC, PsbD, PsbE, PsbH, PsbO, PsbP, PsbR, PsbS), cytochrome F (PetA, PetC, PetE) were significantly down-regulated. However, phosphoenolpyruvate carboxykinase (ATP) (G7KF50, I1JV14, I1J4W3), glyceraldehyde 3-phosphate dehydrogenase (A0A1S2XHT6), fructose diphosphate aldose (I1LJ68, I1NFR3) were significantly up-regulated in photosynthetic carbon fixation pathway. It can be seen that with the increase of drought stress, more photosynthesis-related proteins were inhibited. It can be seen that as the degree of drought stress increased, the more photosynthesis-related proteins were inhibited.

## Discussion

Rubisco is a key enzyme in photosynthetic carbon assimilation, which has a decisive effect on net photosynthetic rate (Pn) [28]. The decrease in plant photosynthesis after drought stress was accompanied by a decrease in the activity of Rubisco and the actual photochemical quantum efficiency. MDH is an important cellular metabolic enzyme that increases under drought stress in response to the high energy demands of plants [29].In this experiment, C_4_ photosynthetic enzyme activity increased after LS, enhancing CO_2_ transfer and fixation, which was used to compensate for the CO_2_ deficit caused by drought stress and may be an adaptive mechanism of liquorice to the drought environment, used to compensate for the metabolic loss of the C_3_ pathway caused by drought [30].

Roots are extremely sensitive to water stress signals and are the first tissue to sense environmental drought stress signals. When the soil dries early, the crop root system is the first to sense the stress and quickly sends out signals so that the whole plant responds to the drought and at the same time adjusts its root form to the soil drought. Li found that a gene encoding a photosystem II polypeptide was up-regulated in wheat roots after drought stress [31].Qin found that drought stress inhibited the root growth of PEPC-transformed wheat, and the decrease in root vigor, root volume and root dry weight became more significant with increasing drought stress [32]. we found that rubisco was significantly down-regulated in roots after LS. Two proteins encoding MDH(A0A1S2XVZ6, A0A072TRS0) were significantly up-regulated in expression after MS. The expression of three proteins (G7KF50, I1JV14, I1J4W3) encoding PEPC(ATP) was significantly up-regulated after SS. It is evident that these enzymes are differentially expressed in different tissues.

The ability of the root system to absorb soil water is key to plant adaptation to drought stress [33]. When the root system senses drought stress, it immediately generates an electrochemical signal to the aboveground tissues, which induces the leaf stomata to close or close in the shortest possible time, thus reducing water loss due to transpiration [34]. In addition, the root responds to the water environment through its own morphology and structure [35, 36]. Previous studies have shown that drought stress significantly affects the accumulation and distribution of plant biomass [37], and the root-shoot ratio is often considered as as a good indicator in the evaluation of drought resistance in plants [38]. As the evaporation rate is high at the surface of the soil layer, it dries more quickly [39]. Therefore, plant investment in deep root growth is a strategy to improve water uptake efficiency and for adaptation to drought conditions, contributing to more efficient water use and improved water distribution in tissues [40]. Our experimental results revealed that with an increase in drought stress the aboveground fresh weight, root fresh weight, plant height and root length all showed an increasing trend followed by a decreasing trend, while the root-shoot ratio showed a gradual increase. In the SS treatment, the aboveground fresh weight, root weight and root length were significantly lower than in the control, while the root-shoot ratio was significantly higher than the control. This indicates that under drought stress, the plant has a strong biomass allocation mechanism and allocates more dry matter preferentially to the roots to improve the root-shoot ratio. Meanwhile, we found that after moderate drought stress, the expression of DNA unwinding enzyme (A0A097PJS6) was significantly down-regulated, and the expression of one protein encoding a subunit of dna-directed RNA polymerase (A0A0R0GY74) and two proteins encoding DNA ligases (A0A1S2YKA4, G7JDY9) were also reduced. This indicates that drought stress affected DNA replication to the extent that it inhibited root elongation.

Sugar metabolism plays an important role in metabolism of living organisms [41]. Sugar is an important assimilation product of plant photosynthesis, and its anabolic and operational distribution directly affects plant growth and development as well as crop yield and quality formation. Soluble sugar is also involved in plant stress resistance as an important osmoregulatory substance. The results of this experiment showed that the main sugar metabolic pathways enriched by DEPs were galactose metabolism, TCA cycle, glycolysis/gluconeogenesis, pentose phosphate pathway, fructose and mannose metabolism, and pentose and glucuronide (Table S3). Compared with the control, the main enrichment pathways of DEPs in the LS treatment were galactose metabolism and the TCA cycle, and the majority of proteins did not change; while more DEPs were significantly up-regulated in the MS and SS treatments, and were enriched in galactose metabolism, TCA cycle, glycolysis/gluconeogenesis, pentose phosphate pathway, fructose and mannose metabolism, pentose and glucuronide interconversion and starch and sucrose metabolism. Amongst these, the most highly expressed DEPs were upregulated in glycolysis/gluconeogenesis, galactose metabolism, starch and sucrose metabolism pathways, mainly pyruvate phosphate hydratase (I1JPW5, I1NAI7), glucose phosphate translocase (I1LDX0), fructose diphosphate aldolase (I1NFR3), ATP-dependent fructose 6-phosphate kinase (A0A1S2Z109, I1J4F8, A0A1S2Y6J6), pyruvate kinase (A0A0R4J2N4, A0A072VPP9, Q1SN32, A0A1S2YIN3), and alcohol dehydrogenase 1 (G7J5M6). Also, the expression of isoamylase (A0A1S2XS14) was significantly upregulated in the MS treatment, which may promote starch hydrolysis, while significant expression was not observed in the SS treatment. This indicates that MS increased the sugar metabolism activity of the root.

Raffinose, an oligosaccharide unique to plants, consists of a series of α-1,6-galactosyl extensions to sucrose, and its content in plants is second only to sucrose. Cottonseed synthetase catalyzes the synthesis of cottonseed sugar from inositol galactosides and sucrose. In the presence of β-amylase, starch can be hydrolyzed to maltose, which is then converted to sucrose in the presence of dismutase [42, 43], and sucrose can be further synthesized into cottonseed sugar and hydrosucrose [44]. It is known that cottonseed sugar synthase gene expression is upregulated in pea seedlings after dehydration treatment and that roots accumulated cottonseed sugar [45]. Expression of At RS5 was induced in *Arabidopsis* under low temperature and drought stress [46]. This indicates that cottonseed glycosylase activity is associated with plant drought resistance.

The results of this experiment showed that cottonseed sugar synthase (I1JND9) was significantly up-regulated after drought stress, catalyzing the synthesis of cottonseed sugar from sucrose; phosphoglucose translocase (I1LDX0), ATP-dependent fructose 6-phosphate kinase (A0A1S2Z109, I1J4F8, A0A1S2Y6J6) and cottonseed sugar synthase (B2ZF65) were significantly upregulated in the MS treatment, promoting α-D-glucose-6P and cottonseed sugar synthesis; cottonseed sugar synthase 3 (B2ZF65, G7JFC4), galactitol-sucrose galactosyltransferase 6 isoform X1 (A0A1S2YQS8), hydrastase (A0A1S2XV57) and α-galactosidase (I1N3A6) which were significantly upregulated in the SS treatment( Table S3), promoting galactitol and sucrose synthesis into galactose and glucose, and increasing the soluble sugar content in the body.

In conclusion, drought resistance was improved mainly by the accumulation of cottonseed sugars in the LS and MS treatments, and was increased mainly by galactose and glucose in response to drought stress in the SS treatment. In addition, galactose metabolism responded faster to water stress, and in addition to that, regulation of fructose and mannose metabolism played an important role in response to greater levels of drought stress.

Aldehydes are intermediates of several metabolic pathways, and excessive accumulation of aldehydes can lead to genetic toxicities, such as chromosomal aberrations and protein inhibition, while aldehyde dehydrogenase (ADLH) can catalyze the oxidative dehydrogenation of aldehydes to the corresponding carboxylic acids, which can alleviate the toxicities of aldehydes [47]. In this study we found that aldehyde dehydrogenase was significantly and consistently upregulated in the fatty acid degradation pathway after drought stress. We hypothesize that liquorice roots reduce aldehyde damage by upregulating the expression of aldehyde dehydrogenase, resulting in increased resistance to drought conditions. Lipoxygenase has an important role in plant responses to stress [48], and its oxygenation reaction catalyzes the conversion of polyunsaturated fatty acids (PUFA) to hydroperoxides. In this study, lipoxygenase (A7LCD5, I1KH70, A0A1S2YZ86, G7IS29) was significantly upregulated in the linoleic acid metabolic pathway in the LS and MS treatments, while it was not significantly enriched after SS(Table S3). Meanwhile, DEPs were mainly enriched in the unsaturated fatty acid biosynthesis and fatty acid metabolism pathways in the MS and SS treatments. In conclusion, in the MS and SS treatments, drought resistance of liquorice was enhanced by upregulation of lipoxygenase catalyzing the oxidation of unsaturated fatty acids that provided energy for metabolic processes, and by reducing the accumulation of aldehydes through upregulation of aldehyde dehydrogenase.

Free polyimine degraded by diamine oxidase and polyamine oxidase can promote accumulation of proline through the production of γ-aminobutyric acid. P5C5 plays an important role in biosynthesis of proline, and overexpression of P5C5 in transgenic plants promotes production of proline synthase; in experiments transgenic plants synthesized 10–18 times more proline than control plants [49]. Furthermore, transgenic rice overexpressing P5CS has significantly higher tolerance to salinity and water stress [50]. In our study, the greatest number of DEPs were enriched in the biosynthetic pathway of amino acids. Meanwhile, expression of delta-1-pyrroline-5-carboxylate synthase (P5C5, A0A072UH55) was consistently up-regulated after drought stress, indicating that its role in the process of drought stress is not trivial. In the ‘alanine, aspartate and glutamate metabolism’ pathway, glutamate dehydrogenase (I1J9Q7) was upregulated in both the LS and MS treatments, promoting NH_3_ accumulation. In contrast, in the SS treatment, pyruvate synthesis (via glycolysis/gluconeogenesis and up-regulation of pyruvate dehydrogenase) was promoted by up-regulated expression of alanine-glyoxylate transaminase (A0A0R4J2K3), alanine-glyoxylate aminotransferase 2 homolog 3 (A0A1S2XPL6) and alanine-glyoxylate aminotransferase (G7L4C3) ( Table S3). Interestingly, in the arginine biosynthesis and proline metabolism pathways, arginase (I3SM21) and argininosuccinate lyase (I1JV71) were up-regulated in expression in the LS treatment, promoting arginine synthesis, while ornithine decarboxylase (Q70MR6) was up-regulated in expression in the MS treatment, promoting conversion of ornithine to putrescine; ornithine decarboxylase (ornithine decarboxylase, ODC, Q70MR6) was down-regulated and inhibited polyamine biosynthesis in the SS treatment. S-adenosy-L-methionine synthetase (SAMS) is an important rate-limiting enzyme in polyamine synthesis [51], catalyzing production of thiosemicarbazone (SAM) from methionine and ATP. SAM is involved in a variety of biochemical reactions and is an important metabolic product, being the only methyl donor for methylation reactions in plant alkaloid synthesis and a precursor of polyamines in plants [52]. Polyamines, as secondary metabolites in the metabolic process of organisms, have important roles in improving plant stress resistance, regulating plant growth and development, delaying senescence, and controlling morphogenesis [53]. In the present study, SAMS was involved in amino acid biosynthesis, cysteine and methionine metabolism, secondary metabolite biosynthesis and metabolic pathways annotated to and down-regulated in in the MS and SS treatments. In conclusion, the proline synthesis pathway was activated in the LS treatment, leading to an increase in proline content in response to drought stress. In the MS treatment SAMS and other enzymes were significantly down-regulated which inhibited the synthesis of polyamines.

Interactions between plants and the environment are mainly mediated by synthesized secondary metabolites [54]. Phenylpropane is a precursor of versatile phenolic compounds in plants that are involved in stress response in plant cells [55]. The first step in the biosynthesis of ubiquinone and other terpenoid quinones is the generation of 4-coumaric acid from trans-cinnamic acid ester catalyzed by trans-cinnamic acid 4-monooxygenase, which is hydroxylated by 4-coumaric acid-coenzyme A ligase (4Cl) to generate β-coumaric acid-coenzyme A, which is involved in the flavonoid biosynthetic pathway [56]. Meanwhile, 4Cl plays an important role in plant biosynthesis and affects the accumulation of phenylpropane in plants [57]. In this study, some DEPs were found to be enriched in a large number of secondary metabolism-related pathways after drought stress, including flavonoid biosynthesis, terpene skeleton biosynthesis, carotenoid biosynthesis, sesquiterpene and triterpene biosynthesis, and phenol propane biosynthesis ( Table S3). In the phenylpropane biosynthesis pathway, phenylalanine aminolytic enzyme (PAL, I1NA96) was significantly down-regulated in the MS and SS treatments, which may result in reduced trans-cinnamic acid synthesis and accumulation of phenylalanine. Among them, trans-cinnamic acid 4-monooxygenase (CYP) was significantly up-regulated in the MS treatment, promoting 4-coumaric acid synthesis. In the SS treatment, 4Cl and cinnamoyl coenzyme A reductase (I1KGT7, I1M4H6) acted to synthesize cinnamic aldehyde and improve plant resistance. 3-Hydroxy-3-methylglutaryl coenzyme A synthase and 1-deoxy-D-xylulose-5-phosphate synthase are the first rate-limiting enzymes of the mevalonate (MVA) and deoxyxylulose-5-phosphate (DXP) or methylerythritol-4-phosphate (MEP) pathways, respectively, and are used as raw materials for the synthesis of isopentenyl (IPP) and dimethylallyl diphosphate (DMAPP) from pyruvate and glyceraldehyde-3-phosphate in plastids, which are the precursor five-carbon compounds of the terpenoid biosynthetic pathway. In this experiment, 3-hydroxy-3-methylglutaryl coenzyme A synthase was significantly up-regulated in the MS treatment and significantly down-regulated in the SS treatment, and 3-hydroxy-3-methylglutaryl coenzyme A reductase (K7LEW4) and 1-deoxy-D-xylulose-5-phosphate synthase were significantly down-regulated in the SS treatment, which inhibited the synthesis of terpene precursor pentacarbons. Squalene synthase (SQS) is involved in the early stages of glycyrrhetinic acid biosynthesis and encodes farnesyl diphosphate (FPP), which provides a direct precursor for the formation of the triterpene (glycyrrhetinic acid) skeleton [23]. β-AS catalyzes 2,3-oxidation of squalene to produce β-amyrin, a key step in the formation of the triterpene skeleton [25]. Nasrollahi et al. reported that drought stress up-regulated expression of key genes involved in triterpenoid saponin biosynthesis in liquorice and increased the concentration of glycyrrhetinic acid [23]. In this study, we found that SQS (Q8GSL6) was consistently up-regulated after drought stress, leaving squalene synthesis unaffected, while squalene monooxygenase (A0A1S2YI26) was significantly down-regulated in the MS treatment, affecting squalene 2,3-epoxide synthesis. Furthermore, β-coumarin synthase (W5XM28, A0A0R0IYV7, Q84PE3) was also significantly down-regulated in the SS treatment, which inhibited β-coumarin synthesis. We suggest that, at the protein level, in the SS treatment down-regulation of proteins was related to synthesis of terpene precursors of five-carbon compounds (e.g. squalene monooxygenase and β-coumarinol synthase), which in turn inhibits the synthesis of terpene precursors of five-carbon compounds and ultimately leads to a decrease in glycyrrhetinic acid content.

Ribosomal proteins are important structural components in protein synthesis and play an important role in protein synthesis [58]. Up-regulation of ribosomal protein abundance helps plants to enhance resistance to stress [59]. In this experiment, one ribosomal protein (A0A1S2Y4A2) was significantly down-regulated in the LS treatment compared with the control, and none of the other proteins related to protein synthesis were significantly changed, while three proteins were significantly up-regulated in the MS treatment (A0A1S2X8K2; A0A072VMH3; B7FGL9), and protein (A0A1S2Y4A2) remained significantly down-regulated in the SS treatment, while two proteins were significantly up-regulated (A0A1S2X8K2; I1M223). In the LS and MS treatments, 60S ribosomal protein (A0A1S2Y4A2) was significantly down regulated and inhibited protein synthesis in the root system. In contrast, expression of 40S ribosomal protein (A0A1S2X8K2) was significantly upregulated in the MS and SS treatment, indicating that this type of protein synthesis has an important role in the response of liquorice to drought( Table S3). This indicates the strong adaptive regulation ability of liquorice under drought stress.

Heat-shock proteins (HSPs)/chaperones, which act as buffers to limit misfolding, assist protein refolding and stabilizing, and are also linked to signaling under stressful conditions [60]. Under stress, the increase in reactive oxygen species (ROS) triggers synthesis of HSP70, which plays a key role in preventing aggregation of unnatural proteins and assisting their reversion [61], further enhancing the activity of antioxidant enzymes and improving organismal resistance [62]. HSP90 has important roles in protein folding, signal transduction networks, cell cycle control, protein degradation and cellular protein transport. In addition, as chaperones, small heat stress proteins (SHSPs) protect proteins from aggregation under stress conditions and have enzymatic activities essential for regulating plant growth and metabolism [63], thus improving cellular resistance to adversity [64]. In this study, a significant amount of HSPs were expressed in the ‘protein processing in the endoplasmic reticulum (PPER)’ pathway, in which HSP20 family protein (A0A0R0FH01) and HSP60 family protein (B7FLW6) were upregulated in the LS treatment, while HSP70 family proteins (A0A072V683, A0A076KWI9) were down-regulated in the MS treatment; HSP10 family proteins (C6SXN0), HSP20 family proteins (G7J8A4, A0A0R0FH01), HSP60 family proteins (I1MJ28, Q1RSH4, I1LAL4, I1N5D5, A0A1S2YGW8, B7FLW6, I1KGB8), HSP70 family proteins (A0A396J978, A0A076KWI9 and HSP90 family proteins (A0A1S2Y850) were up-regulated. In the SS treatment HSP20 family proteins (C6T2N6, A0A0R0FH01, A0A072VLZ3, G7J8A4, P30236 (22.0 kDa), A0A1S2XM95 (15.7 kDa)), HSP60 family proteins (I1MJ28, Q1RSH4, B7FLW6), HSP70 family (G7JFK1, I1MT10, A0A396J978, A0A396J4, A8A0A076KWI9, A0A396JAW6, G7ZZY8), and HSP90 family proteins (A0A1S2Y850) were up-regulated( Table S3). In conclusion, these data suggest that protein processing is affected by drought stress. In particular, HSP20 and HSP70 accumulation helps protein refolding, stabilization and is associated with signal transduction.

Under drought stress, LEA proteins are bound to the inner membrane system as dehydration protectors with high water retention capacity, which can stabilize their structure and protect biomolecules, thus reducing water dissipation. The results of this experiment showed that the expression of LEA_2 domain-containing protein (C6TMR7, I1L9J7) was higher than the control after light and moderate drought stress, but lower than the control after severe drought stress, and the expression of a protein encoding LEA (A0A072V5S0) was significantly down-regulated after severe drought stress.

AQP facilitates water transport across the membrane inside and outside the cell by reducing the resistance encountered in water transport across the membrane, thus promoting faster water migration down the water potential gradient between cells. In this experiment, the expression of the vesicle membrane intrinsic protein TIP (I3SHN0) was significantly down-regulated after drought stress, and the expression of the Major intrinsic protein (MIP) family transporter (Q6J1S8) was significantly down-regulated after severe drought stress, presumably, after drought stress, liquorice roots helped to maintain the water content in plant roots by down-regulating the expression of AQP. However, cell integrity may be disrupted.

As drought stress increases, ABA is synthesized in the root system, and the root system produces a root signal ABA due to water loss, which is transported to the leaves and stems with the xylem, so that stomatal opening is reduced or inhibited [65]. In this experiment, ABA-responsive protein (G7IMZ3), protein C2-DOMAIN ABA-RELATED 9-like (A0A1S2Y3L2), and protein C2-DOMAIN ABA-RELATED 7 (A0A1S2YIG9) were significantly up-regulated in expression after severe drought stress; also, ABA-responsive protein (G7IMZ3) was significantly up-regulated in expression.

## Conclusion

The root system is an important organ for absorption of water and nutrients to support above-ground organs. Under water stress, plant roots are involved in drought-related mechanisms by adjusting various metabolic substances, to induce the expression of relevant drought-resistant genes and enzymes to resist water stress. In this study, 837 differentially expressed proteins (DEPs) were identified in liquorice roots after different drought stresses using TMT sequencing. Functional analysis revealed that liquorice roots exhibited specific responses to different drought stresses. In the LS treatment accumulation of osmolytes such as cottonseed sugar and proline was the mechanism for improving resistance in liquorice. In the MS and SS treatments, oxidation of unsaturated fatty acids, as well as glucose and galactose accumulation were promoted in response to drought stress. It was also found that synthesis of terpene precursors, pentacene 2,3-epoxide and β-coumarin, was inhibited and the accumulation of triterpenoids (glycyrrhetinic acid) was affected in the MS and SS treatment.

**Fig. 8:**
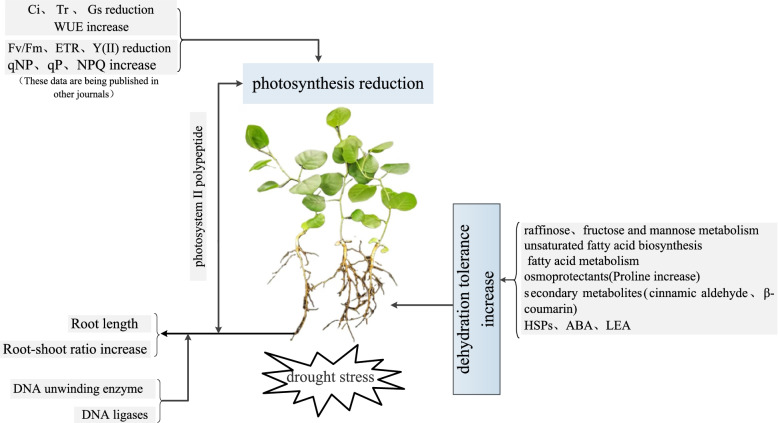
Response model of liquorice under drought stress. Note: Ci: intercellular CO_2_ concentration, Tr: transpiration rate, Gs: stomatal conductance, WUE:water use efficiency, ETR:electron transfer rate, Y(II):effective quantum yield of photosystem II

In summary, we propose a model for the response of liquorice under drought stress (Fig. 8). The net photosynthetic rate of liquorice leaves was reduced and photosynthesis-related enzymes were changed to varying degrees after the stimulation of liquorice by drought stress. At the same time, the root growth of liquorice was stunted, but the root-shoot ratio and water use efficiency increased. Sugar metabolism, lipid metabolism, amino acid metabolism, secondary metabolism and drought response proteins in roots induced increased resistance in liquorice roots. We hypothesize that photosynthetic peptides in leaves after drought stress regulate root growth through long-distance transport to the roots. In conclusion, these responses work together to confer different drought resistance to liquorice.

## Material and methods

### Plant culture and drought treatments

Liquorice seeds were purchased from Beijing Shizhen Chinese Herbal Technology Co. Seeds with intact kernels were selected, surface sterilized in 80% concentrated sulfuric acid for 10 min, rinsed several times in tap water, and twice in distilled water, and placed in Petri dishes for germination. After seedling emergence, they were grown in compost in pots(Height 20 cm, diameter 22 cm) under normal management practices (80% relative soil moisture content per day, 10 h of artificial light supplementation per day). When the seedlings grew to 4–5 true leaves the number in each pot was reduced to five for uniform growth. Healthy seedlings were maintained in each pot and used in experiments after 2 months of growth.

There were four treatments: normal water supply control (CK), light stress (LS), moderate stress (MS) and severe stress (SS); there were ten replicate pots per treatment. The relative soil water content (SRWC) of the four treatments was 80% ± 3%, 65% ± 3%, 50% ± 3% and 35% ± 3%, respectively. Natural drought was maintained in the different treatment levels, and soil moisture content was controlled by weighing and replenished the water at 18:00 every day to maintain different drought stress levels. Drought stress treatments were applied for 10 days, after which samples were taken between 10:00 and 11:00 a.m. on the last day prior to storage and evaluation of relevant indices.

### Measurement of photosynthetic enzyme activity

Take 0.1 g of leaf, cut it up quickly on ice and put it into a pre-cooled grinding tube, add 1 ml of enzyme extract and put it into a low temperature grinder (60 Hz, 20 min), after grinding, put it into a low temperature centrifuge (4 °C, 12,000 r/min, 30 min), the supernatant is the enzyme activity to be measured. ribulose 1,5-diphosphate carboxylase/oxygenase (EC4.1.1.39, Rubisco), pyruvate orthophosphate dikinase (EC 2.7.9.1, PPDK), phosphoenolpyruvate carboxylase(EC4.1.1.31, PEPC)and NADP-Malate dehydrogenase (EC1.1.1.82, NADP-MDH) were determined using kits from Suzhou GRS Biotechnology Co. The assay was performed according to the kit instructions.

### Total protein extraction from liquorice root

Total liquorice root protein was extracted using standard methods [66]. A subsample of liquorice root tissue was ground to a powder in a grinder (purchased from Shanghai Jingxin/ 24-well), at low temperature, quickly transferred to a liquid nitrogen pre-cooled centrifuge tube, and an appropriate amount of protein lysis solution (4% SDS, 10 m M DTT, 100 m M TEAB) was added, mixed and sonicated for 5 min then incubated at 95 °C for 8 min, followed by centrifugation at 4 °C and 12,000 g for 15 min. The supernatant was incubated with 10 m M DTT at 56 °C for 1 h. After that, iodoacetamide (IAM) was added in sufficient quantities and the reaction allowed to proceed at room temperature for 1 h. The precipitate was precipitated by adding 4 times the volume of acetone at -20 °C for 2 h. The precipitate was collected by centrifugation at 12,000 g for 15 min at 4 °C. The precipitate was then resuspended and washed with 1 m L of pre-cooled acetone, centrifuged at 4 °C for 15 min at 12,000 g, collected, air dried and solubilized by adding the appropriate amount of proteolytic solution (8 M urea, 100 m M TEAB, pH = 8.5).

Using a Bradford protein quantification kit [67], BSA standard protein solution was prepared in a concentration gradient of 0–0.5 µg/µL according to the instructions; different gradients of BSA standard protein solution and different dilutions of the sample to be tested were placed in a 96-well plate, at a volume of 20 µL. To these 180 µL of G250 staining solution was added rapidly to each well and incubated at room temperature for 5 min before measuring the absorbance at 595 nm. The absorbance of the standard protein solution was used to draw a standard curve and calculate the protein concentration of the samples to be measured.

### TMT labeling and peptide separation

Proteolytic solution was added to each protein sample (100 μL), trypsin was added and 100 m M TEAB buffer, mixed well and then digested at 37 °C for 4 h. More trypsin was added and CaCl_2_ to digest overnight. Formic acid was then added to adjust the pH to less than 3, mixed well and centrifuged at 12,000 g for 5 min at room temperature. The supernatant was slowly passed through a C18 column for desalting, and the filtrate collected and vacuum freeze dried. To each 100 μL of 0.1 M TEAB buffer was added to dissolve the peptide and 41 µL of TMT labeling reagent, mixed well, and the reaction allowed to proceed at room temperature for 2 h. Afterwards, 8% ammonia was added to terminate the reaction, mixed with an equal volume of labeled sample, desalinate, and freeze-dry under vacuum [68].

The sample was mixed, desalted and freeze-dried under vacuum. The sample was fractionated on a Waters BEH C18 column (4.6 × 250 mm, 5 μm) using an L-3000 HPLC system with the elution gradient shown in Table S4. One tube was collected every minute and divided into ten fractions, which were lyophilized and dissolved by adding 0.1% formic acid to each.

### LC–MS/MS analysis and protein identification

The liquid chromatography elution conditions were as shown in Table S5. For each supernatant 1 μg of each fraction was taken for mass spectrometry analysis. The mass spectrometry parameters were set as follows:

The spray voltage was 2.3 kV, the temperature of the transfer tube was 320 °C, the resolution of the primary mass spectrum was set to 60,000 (200 m/z), the resolution of the secondary mass spectrum was set to 45,000 (200 m/z), the mass spectrum was acquired in data-dependent mode, the threshold intensity was set to 1.2 × 105, the maximum injection time was 86 ms and the dynamic exclusion range was set to 20 s. The raw mass spectrometry data were generated (.raw). The raw data obtained by LC–MS/MS were searched using the search engine Proteome Discoverer 2.4 (PD2.4, Thermo).

The database was queried with the Glycyrrhiza transcriptome self-built database (717,789-X101SC20032527-Z01-Glycyrrhiza uralensis fasta (206,560 sequences)) for comparison. Differentially expressed proteins (DEPs) were defined according to p < 0.05 (Student t-test method) and the absolute value of the fold change (FC) of the protein was greater than 1.5 (up-regulated protein: FC > 1.5; down-regulated protein: FC < 0.67), and the protein was considered to have undergone a significant change if the above conditions were met [69].

### Protein bioinformatics analysis

Gene Ontology (GO, http://www.geneontology.org) functional annotation and classification were performed for the proteins in the experiments using interproscan software [70]. Protein functions were classified by acquiring entries and sub-category terms, including molecular function (MF), biological process (BP) and cellular component (CC), by Fisher's Exact Test. The GO entry was considered significantly enriched when *P* < 0.05 by Fisher's Exact Test [71]. These proteins were further annotated using the Kyoto Encyclopedia of Genes and Genomes (KEGG, http://www.genome.jp/kegg/) functionally, and the pathway was considered significantly enriched by Fisher's Exact Test at *p* < 0.05, proteins were significantly enriched [72].

### Data analysis

Data were analyzed using Microsoft Excel 2010 and SPSS 20.0 statistical packages. GraphPad Prism 8(GraphPad software Inc., La Jolla, CA, USA) was used for graphical presentation of the data. A factorial experiment based on randomized complete block design was carried out with three replicates (n = 3). Duncan’s multiple range test (*p* < 0.05) was used to compare the means. Proteomic analyses were performed in three biological replicates. Volcano map analysis, cluster heat map analysis and pathway enrichment analysis for GO, IPR and KEGG were performed for DEP [73].

## Supplementary Information

**Additional file 1.**

**Additional file 2.**

**Additional file 3.**

**Additional file 4.**

**Additional file 5.**

## Data Availability

The mass spectrometry proteomics data have been deposited to the ProteomeXchange Consortium (http://proteomecentral.proteomexchange.org) via the iProX partner repository with the dataset identifier PXD033656. The other datasets supporting the conclusions of this article are included within the article and its additional files.

## Abbreviations

DEP: Differentially expressed protein
FDR: False discovery rate
GO: Gene Ontology
KEGG: Kyoto Encyclopedia of Genes and Genomes
LC–MS/MS: Liquid chromatography electrospray ionization tandem mass spectrometry
TMT: Tandem Mass Tag
